# The Puzzle of Aspirin and Iron Deficiency: The Vital Missing Link of the Iron-Chelating Metabolites

**DOI:** 10.3390/ijms25105150

**Published:** 2024-05-09

**Authors:** George J. Kontoghiorghes

**Affiliations:** Postgraduate Research Institute of Science, Technology, Environment and Medicine, Limassol 3021, Cyprus; kontoghiorghes.g.j@pri.ac.cy; Tel.: +357-26-272-076

**Keywords:** aspirin, iron deficiency, iron chelating metabolite, salicylic acid, salicyluric acid, genticic acid, 2,3-dihydroxybenzoic acid, elderly population, drug metabolism, pharmacology

## Abstract

Acetylsalicylic acid or aspirin is the most commonly used drug in the world and is taken daily by millions of people. There is increasing evidence that chronic administration of low-dose aspirin of about 75–100 mg/day can cause iron deficiency anaemia (IDA) in the absence of major gastric bleeding; this is found in a large number of about 20% otherwise healthy elderly (>65 years) individuals. The mechanisms of the cause of IDA in this category of individuals are still largely unknown. Evidence is presented suggesting that a likely cause of IDA in this category of aspirin users is the chelation activity and increased excretion of iron caused by aspirin chelating metabolites (ACMs). It is estimated that 90% of oral aspirin is metabolized into about 70% of the ACMs salicyluric acid, salicylic acid, 2,5-dihydroxybenzoic acid, and 2,3-dihydroxybenzoic acid. All ACMs have a high affinity for binding iron and ability to mobilize iron from different iron pools, causing an overall net increase in iron excretion and altering iron balance. Interestingly, 2,3-dihydroxybenzoic acid has been previously tested in iron-loaded thalassaemia patients, leading to substantial increases in iron excretion. The daily administration of low-dose aspirin for long-term periods is likely to enhance the overall iron excretion in small increments each time due to the combined iron mobilization effect of the ACM. In particular, IDA is likely to occur mainly in populations such as elderly vegetarian adults with meals low in iron content. Furthermore, IDA may be exacerbated by the combinations of ACM with other dietary components, which can prevent iron absorption and enhance iron excretion. Overall, aspirin is acting as a chelating pro-drug similar to dexrazoxane, and the ACM as combination chelation therapy. Iron balance, pharmacological, and other studies on the interaction of iron and aspirin, as well as ACM, are likely to shed more light on the mechanism of IDA. Similar mechanisms of iron chelation through ACM may also be implicated in patient improvements observed in cancer, neurodegenerative, and other disease categories when treated long-term with daily aspirin. In particular, the role of aspirin and ACM in iron metabolism and free radical pathology includes ferroptosis, and may identify other missing links in the therapeutic effects of aspirin in many more diseases. It is suggested that aspirin is the first non-chelating drug described to cause IDA through its ACM metabolites. The therapeutic, pharmacological, toxicological and other implications of aspirin are incomplete without taking into consideration the iron binding and other effects of the ACM.

## 1. Introduction

Iron is one of the essential metal ions and nutrients, and plays an important role for normal physiological functions and maintenance of healthy living in humans. Iron deficiency or overload, as well as other abnormalities of iron metabolism, may cause serious diseases which require treatment for the restoration of iron balance and normal bodily function [1,2,3,4,5,6].

Iron is an integral part of many enzymes and other proteins, as well as a modulator of transcription factors and other co-factors, which ensure the normal function, growth, and development of the human body. Iron also plays a major role in essential physiological processes such as oxygen transport, storage, and utilisation; electron transport; respiration; and other processes involving the metabolism of most natural products such as carbohydrates, lipids, proteins, and nucleic acids, as well as xenobiotic molecules, including drugs [1,2,3,7,8].

The health implications from changes in iron balance and homeostasis are enormous, considering that related diseases such as iron deficiency affect more than a quarter of the world’s population [2,3,7,9,10,11,12,13,14,15] and iron overload in idiopathic haemochromatosis affects one in ten persons of the Caucasian population [2,3,16,17,18,19,20]. Similarly, other iron-related diseases include the haemoglobinopathies, which are the most common group of genetic disorders in humans, with one of them being thalassaemia, identified as the disease with the highest morbidity and mortality rate related to iron or other metal toxicity worldwide [21,22,23,24,25].

Under normal physiological conditions, iron balance can be maintained despite the differences in iron requirements in otherwise healthy individuals. In these cases, the amount of iron absorbed from food is equivalent to the amount of iron excreted or lost from the body [2,7,9,14,15]. However, iron deficiency anaemia (IDA) is a multi-facet disease, which can be caused by many dietary, age, gender, and other factors; in pregnancy and teenagers; and from drugs, traumas, sport, and other activities such as blood donation, vitamin deficiency, malnutrition and different diseases, etc. [9,10,11,12,13,14,15,26,27,28,29,30,31,32,33,34].

The rate of iron absorption in healthy, normal individuals depends mainly in the quantity and quality of iron present in the gastrointestinal tract (GIT). For example, iron deficiency has been widely reported in vegetarian populations, where an insufficient amount of iron is absorbed from vegetarian food containing mostly non-haem iron components, in contrast to meat-containing food, which contains more readily absorbed lipophilic haem iron [7,14,15,28,29,30,31]. Iron deficiency could also be caused through inhibition of iron absorption in the GIT from interactions by many dietary molecules and also orally administered drugs [35,36,37,38,39,40,41,42,43,44]. In particular, the hydrophilic iron-chelating drug deferoxamine is routinely used for the inhibition of iron absorption and in cases of iron poisoning [44,45,46]. In contrast, the lipophilic iron complex of the natural product maltol (ferric iron tri-maltol, or feraccru or accrufer) increases iron absorption and is used for the treatment of IDA [46,47,48].

In contrast to many reports on studies related to the effect of drugs and dietary phytochelators on iron absorption, the effects of drugs on iron excretion have not been fully investigated and are not well documented, except in the case of the iron-chelating drugs deferoxamine, deferiprone, and defarasirox, which are widely used for increasing the excretion of excess iron in iron-loaded patients [7,49,50,51]. The three iron-chelating drugs can also cause iron deficiency in non-iron loaded individuals, as well as zinc deficiency, with other associated side effects following chronic administration [52,53,54,55,56,57,58,59,60]. In almost all the cases of the chelating drugs, natural chelators, and drug metabolites, the available form of iron for chelation and mobilization in humans is protein and non-protein bound and in the ferric state (Fe III) [7,14,43,48,51].

Several clinical investigations have recently reported that the chronic administration of low-dose acetylsalicylic acid or aspirin can cause IDA independent of major bleeding in a large number of elderly, otherwise healthy individuals. The mechanisms and mode of action of aspirin and the cause of IDA in this category of the elderly population have not been identified and are still largely unknown.

Aspirin is one of the most commonly used drugs, which is widely available and inexpensive and can be bought over pharmacy counters or prescribed by physicians. Furthermore, aspirin is one of the oldest drugs and has been in clinical use since 1897, with clinical experience involving different formulations for more than a century [61,62]. It has many uses, including analgesic, anti-inflammatory, antipyretic, and antithrombotic activity [61,62,63]. Different doses are used in general for each condition, including, for example, low doses of about 75–100 mg/day prophylactically for anti-platelet activity and cardiovascular complications, 325 mg or 80 mg twice daily for venous thromboembolism prophylaxis after joint arthroplasty, 300–600 mg every 4–6 h for analgesic function, and 1–2 g every 4–6 h for anti-inflammatory activity with maximum about 2.4–3.6 g/day [64,65,66,67,68]. A low dose of 75–100 mg/day is taken prophylactically by millions of people in the elderly (>60 years) population [64,65,66,67,68]. The main mechanism of action of aspirin is the irreversible inhibition of the iron-containing enzyme cyclo-oxygenase, involved in prostaglandin biosynthesis and platelet aggregation [63,69,70,71].

The pharmacokinetic, metabolic, and therapeutic properties of aspirin can vary between individuals, similarly to other drugs, and depend on absorption, distribution, metabolic, elimination, toxicity (ADMET), genomic, and other characteristics, as well as many factors such as drug dose, drug formulation, GIT dietary content, age and weight of individuals, etc. [72,73,74,75,76,77,78,79,80]. In general, aspirin is rapidly absorbed mainly from the stomach and upper small intestine and mostly hydrolyzed and metabolized to salicylic acid and other metabolites, all of which are excreted in the urine [72,76,81,82].

There is increasing evidence that chronic administration e.g., 3 or more years of low-dose aspirin of about 75–100 mg/day, could cause a 23% higher risk of IDA independent of major bleeding, in a large number of otherwise healthy individuals, usually greater than 65 years of age [83,84,85]. Considering that in addition to millions of normal individuals taking low-dose aspirin for prophylaxis, millions more of patients with cancer, chronic kidney disease, heart failure, diabetes, and many other diseases are also taking low-dose aspirin and are also at risk of IDA. The connection between aspirin use and IDA has not yet been fully characterised. It has recently been suggested that some of the aspirin metabolites have iron-chelating properties (ACM) and may be involved in the cause of IDA [86]. It has also been suggested that the metabolism of aspirin to ACM resembles the cardioprotective chelating pro-drug dexrazoxane, which is metabolized to an EDTA-like chelator [86].

The aim of this review is to examine the iron-binding properties of aspirin, salicylic acid, and other metabolites possessing iron chelating capacity, with emphasis on the prospect of their possible interference regarding iron balance and involvement in the cause of IDA following chronic administration in the aspirin-treated patient populations. Furthermore, it is also intended to examine other factors that may influence iron balance in aspirin-treated individuals, as well as to suggest possible therapeutic and other interventions that may restore iron balance and therapeutic outcomes in these categories of patients, including those with cardiovascular, kidney, cancer, and neurodegenerative diseases.

## 2. Metabolites of Aspirin with Iron Chelating Properties

There are many variations among individuals regarding the pharmacokinetic and metabolic profile of aspirin, and also other variations as a result of the different doses and formulations used [72,76,77,81,82]. Furthermore, several other factors could influence the pharmacokinetic and metabolic parameters of aspirin users, including age, gender, organ function, and interactions with food substances and other drugs [87,88].

In general, orally administered low-dose aspirin of about 75–100 mg is absorbed within minutes from the stomach and upper intestine with about 60–70% bioavailability. Aspirin is rapidly cleared from the circulation with half-life of about 15–20 min [81,82]. Aspirin is mostly metabolized or de-acetylated through hydrolysis, almost exclusively to salicylic acid, the first major metabolite in plasma, predominantly in the liver but also in the stomach, intestine, and blood [87,88]. Salicylic acid has a longer half-life in comparison to aspirin, of about 2–6 h, and is mostly metabolized in the liver, with only about 10% of unchanged salicylic acid being excreted in the urine [69,81,82,87,88,89,90,91]. The metabolites of salicylic acid are salicyluric acid, two glucuronide conjugates (salicylacyl glucuronide and salicylphenol glucuronide), and two dihydroxybenzoic acids (2,5-dihydroxybenzoic acid and 2,3-dihydroxybenzoic acid or 2,3-DHB) [87,88,89,90,91,92,93,94].

All the salicylate metabolites are cleared through the kidneys and excreted in the urine at different concentrations, with salicyluric acid predominating at about 70%, glucuronide conjugates at about 15%, and much lower quantities of dihydroxybenzoic acids at about 2–3% [81,82,87,88,89,90,91]. Many factors affect the level of aspirin and each of its metabolites in plasma following the metabolic transformation of aspirin [90,95,96,97]. The maximum concentration in plasma of aspirin administered at different doses, as well as its metabolites, depends mainly on the dose. The concentration of each of the aspirin metabolites in plasma also depends on many other factors, including, for example, the enzyme activity involved in each step of the metabolic transformation, such as differences between individuals in glucuronidation and the formation of glucuronide conjugates, as previously observed with other drugs [88,98,99].

**Figure 1:**
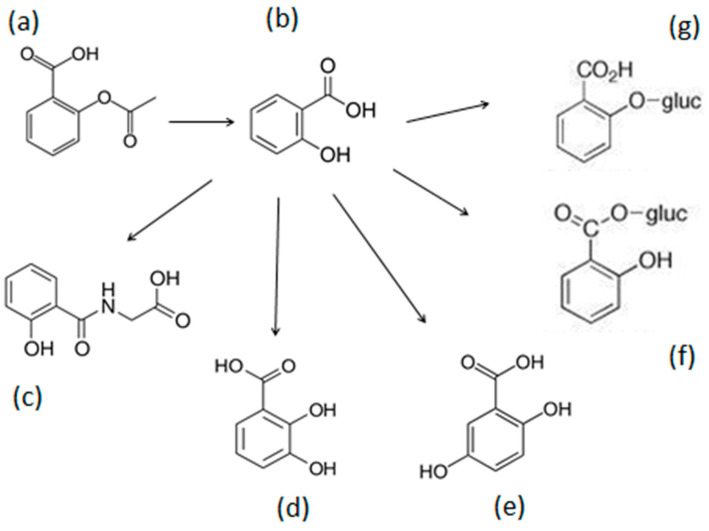
The metabolic transformation of aspirin. Aspirin or acetyl salicylic acid (**a**) is metabolized to salicylic acid (**b**), which is then further metabolized to another five metabolites, which are salicyluric acid, (**c**) 2,3-dihydroxybenzoic acid, (**d**) 2,5-dihydroxybenzoic acid (gentisic acid), (**e**) salicylacyl glucuronide, (**f**) and salicylphenol glucuronide (**g**).

The structural chemical characteristics, including potential iron-binding ligands and sites, are important parameters for the determination of the chelation potential of different drugs, metabolites, or other synthetic and natural molecules [7,14,43,48,51]. The chemical structures of aspirin and its metabolites, namely salicylic acid, salicyluric acid, 2,3-DHB, 2,5-dihydroxybenzoic acid (gentisic acid), salicylacyl glucuronide, and salicylphenol glucuronide, are shown in Figure 1a–g.

Chelators are, in general, organic compounds which possess at least two ligands with electron donor atoms such as O and N, which have affinity for binding metal ions and the appropriate proximity between them to form a coordinated covalent bond and, overall, a ring with the metal ion as the closing member. Within this context, there are many organic biological molecules, including some of the aspirin metabolites possessing electron donor atoms on ligands, which can be involved in metal complex formation. The donor atoms can be present in ligands of acidic groups such as -OH, O=C-OH, -NOH, where the proton can be displaced by the metal ion or in Lewis bases such as -C=O and -NH_2_ [7,14,43,48,51].

In considering the iron-binding capacity of aspirin and its metabolites, it appears from the chemical structures that almost all contain hydroxyl and other ligands with a potential for iron binding. In particular, the principal chelating sites in the aspirin metabolites with the required proximity for chelating iron are oxygen ligands from phenolic (-OH), carboxylate (O=C-OH), and carbonyl (-C=O) groups (Figure 1). Furthermore, an interchange between the iron-binding ligands may also take place in the case of, for example, 2,3-DHB depending on the pH, as previously suggested for similar chelating molecules [99]. In particular, the presence of the 2-hydroxyl (-OH) ligand increases the chelation potential of all metabolites except in the case of salicylphenol glucuronide, where the 2-hydroxyl group is replaced following conjugation with glucuronide, which diminishes the iron-binding potential site of this metabolite (Figure 1g).

**Figure 2:**
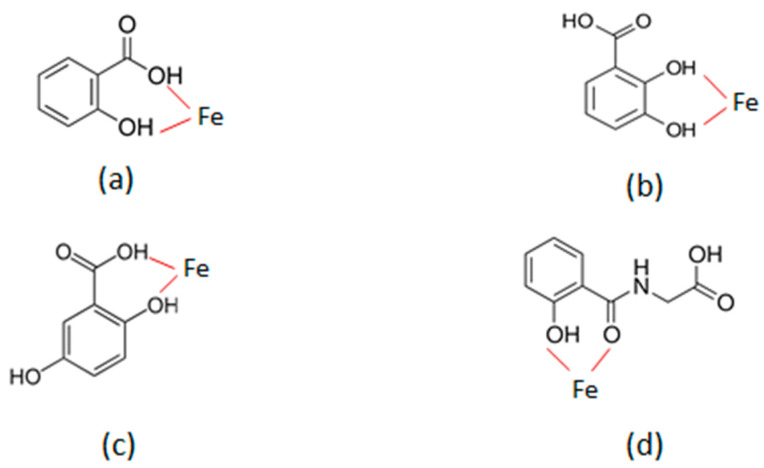
The potential iron-chelating sites of the aspirin metabolites. All the aspirin-chelating metabolites appear to be potential bidentate chelators. The ligands involved in iron (Fe III) binding and the formation of iron (Fe III) complexes are shown for salicylic acid (**a**), 2,3-dihydroxybenzoic acid (**b**), 2,5-dihydroxybenzoic acid (**c**), and salicyluric acid (**d**). A similar iron-binding site of (**d**) is theoretically possible for the salicylacyl glucuronide, both of which are weaker iron chelators in comparison to (**a**) and (**c**) and much weaker than (**b**), which is considered the strongest iron chelator of the aspirin metabolites.

In contrast to salicylphenol glucuronide, the metabolite with the highest iron chelating potential is 2,3-DHB, where the two adjacent hydroxyl ligands are similar to the catechol-containing chelators, such as those found in the bacterial siderophore enterobactin, which is a very powerful iron chelator synthesized by bacteria to scavenge iron from the surrounding environment [43,99,100]. Furthermore, aspirin and most of its metabolites contain a carboxylate metal-binding site similar to EDTA and DTPA, two well-known chelating drugs, and also the naturally occurring chelator citric acid, all of which have different iron binding potential [7,43,51,100]. The potential ligands involved in the iron-chelating binding sites in the case of the four most important ACMs are shown in Figure 2.

Following iron binding, a proton is released from the hydroxyl ligand (-OH) of the phenol group of the ACM, allowing the formation of oxygen-iron bond (-O-Fe) (Figure 2). Similarly, on iron binding a proton is also released from the carboxylate group (O=C-OH) of the ACM allowing the formation of oxygen-iron bond (O=C-O-Fe). In the case of salicyluric acid, iron binding is from the adjacent carbonyl (-C=O) group where a pair of electrons from oxygen (-C=O:) group is used for the bond with iron (-C=O^…^Fe).

In general, it appears from the chemical structural characteristics that all ACM are likely to be mostly bidentate chelators (Figure 2), which potentially can form a 3 chelator:1 iron molar ratio iron comples at physiological pH, with an octahedral structure similar to other bidentate iron chelating drugs, such as deferiprone [7,14,43,51,100]. However, at lower pH levels and low chelator metabolite concentration, 1:1 and 2:1 chelator: iron molar ratio complexes can also be potentially formed by the ACM [100]. Similarly, the distant phenolic (-OH), carboxylate (O=C-OH), and carbonyl (-C=O) groups present in aspirin and metabolites are also potential ligands for binding iron under certain conditions, including in cases of mixed iron complexes [99,100].

The iron-binding properties and effects of some of the ACMs have been previously reported from in vitro, in vivo, and clinical studies. In particular, at least four of the ACMs (2,3-DHB, 2,5-dihydroxybenzoic acid, salicyluric acid and salicylic acid) are known to be natural plant products involved in many other biological processes (Figure 2) [101,102,103].

## 3. In Vitro, In Vivo and Clinical Iron Studies of Aspirin and the Aspirin Metabolites

There are many studies reporting different interactions of aspirin and the aspirin metabolites with iron, and also iron metabolism in general. These interactions are similar to other chelating agents or molecules with iron-binding ligands but of different thermodynamic, kinetic, pharmacological, and toxicological parameters in each case [7,14,51,99,100]. In this section, a summary of some of the iron-binding effects of aspirin and the ACM will be introduced, with emphasis on the implications of iron chelation and iron metabolism.

### 3.1. Interactions of 2,3-Dihydroxybenzoic Acid with Iron

The most widely known and studied ACM for its iron binding properties is the naturally occurring 2,3-DHB [43,99,103]. The iron-binding site of 2,3-DHB, the smallest in production of ACM, are the two adjacent hydroxyl groups, which are involved in the formation of a 3 chelator:1 iron molar ratio iron complex at a physiological pH, similar to other catechol-containing chelators (Figure 2) [99,100]. There are hundreds of investigations, including clinical trials, reported regarding the iron-binding properties and effects of this aspirin metabolite. In particular, 2,3-DHB was selected for clinical use in the treatment of iron overload in thalassaemia patients from a group of 26 benzoic acids following preclinical studies in different animal species [104].

Several clinical investigations have been reported using 2,3-DHB. In one double-blind clinical trial involving 15 regularly red blood cell-transfused thalassaemia patients, the administration of 4 × 25 mg/kg/day of 2,3-DHB for one year resulted in the stabilization of the iron load in the patients [105]. Most importantly, there were no toxic side effects reported during the one-year clinical trial period [105]. In another iron balance clinical study involving 5 iron-loaded thalassaemia patients, 2,3-DHB administered at 25 mg/day for 8 days caused, on average, a 4.5 mg/day increase in iron excretion, while when the dose increased to 4 × 25 mg/kg in 8 patients for 21 days, the iron excretion increased on average to 6.5 mg/day. The drug was well-tolerated, with some mild gastrointestinal complaints [106].

Eventually, further clinical studies on the use of 2,3-DHB in the treatment of transfusional iron overload in thalassaemia were abandoned due to insufficient iron excretion in comparison to the much higher levels of iron intake from regular red blood cell transfusions in most patients [107]. However, although that this natural experimental chelator was not sufficiently effective, its low toxicity may have been useful for its use in combination therapies with other chelating drugs, as previously suggested [108].

More information on the safety and efficacy of 2,3-DHB was obtained from animal studies. In one such study using iron-loaded rats, 2,3-DHB was identified to be effective in mobilizing iron, without causing increases in calcium, magnesium, copper, or zinc excretion [109]. Further toxicity studies have shown that the LD50 for oral 2,3-DHB is more than 3 g/kg in rabbits, while chronic administration of 600 mg/kg in mice and rats has shown no signs of toxicity [109]. Furthermore, its oral administration caused a decrease in radiolabelled (Fe 59) iron absorption in rats, but in another study and under different conditions, a small increase in radiolabelled (Fe 59) absorption and body iron intake in mice was reported [46,109].

There are also many cell, protein, and other in vitro studies involving 2,3-DHB regarding its role as an iron chelator, natural siderophore, and an antioxidant [110,111]. For example, in cell studies, it has been shown to mobilize iron from ferritin, while in transferrin studies, iron could not be mobilized, but instead 2,3-DHB appeared to act as a synergistic anion in the iron binding site of transferrin [112,113]. In different cell studies, no cytotoxic effects on neuroblastoma cells have been shown by 2,3-DHB, while in studies on red blood cells, 2,3-DHB was not effective in transporting iron across the cell membrane, an activity facilitated mostly by other lipophilic chelators such as maltol [100,114,115].

The antioxidant activity of 2,3-DHB has been studied in different models of oxidative stress toxicity, where iron chelation has been considered as the major mechanism of the prevention of oxidative damage [116,117]. For example, the antioxidant effects of 2,3-DHB were examined in free radical toxicity models of the breakdown of deoxyribose, IgG damage due to UV irradiation, and muscle tissue damage due to homogenization. In these studies, the level of antioxidant activity of 2,3-DHB was proportional to its concentration, and usually, levels greater than 0.1 mM appeared to be effective for antioxidant activity in these three different models [118].

In the meantime, many clinical and in vivo studies examining the antioxidant effects of 2,3-DHB in several diseases have been carried out using aspirin as a pro-drug. In particular, increased production of 2,3-DHB and 2,5-dihydroxybenzoic acid was anticipated from aspirin metabolism in conditions with increased production of hydroxyl radical during oxidative stress [119,120,121]. In many cases, the increased production of these two ACMs, and especially 2,3-DHB, was implicated in the increased antioxidant effects observed during the administration of aspirin in inflammatory and other similar conditions [117].

Overall, 2,3-DHB appears to be a generally safe and an effective iron chelator for increasing iron excretion, and to possess high antioxidant activity.

### 3.2. Interactions of Salicylic Acid with Iron

There is wide interest in the properties and effects of salicylic acid as a natural plant product and dietary component, and as a first major metabolite of aspirin, not only in the context of therapeutic activity but also in different pharmacological, toxicological, ecological, and other aspects related to medicine [101,122,123,124,125,126,127]. In this section, only aspects related to iron will be discussed, despite the fact that the interaction of salicylic acid with iron and the formation of the iron-salicylate complex affects all these and related activities. 

Salicylic acid was identified as an iron chelator in about the early 1950s and studied especially for its high iron-binding avidity, including the prospect of its clinical use, as well as its derivatives, in the treatment of transfusional iron overload in thalassaemia [128].

The iron binding site of salicylic acid consists of a carboxylate ligand and an adjacent hydroxyl ligand, as shown in Figure 2. It forms 3 chelator:1 iron molar ratio iron complexes at physiological pH, which are of an octahedral structure, and lower molar ratio chelator:iron complexes at low salicylate concentrations, similar to 2,3-DHB and other bidentate iron-chelating drugs [128,129,130,131].

There are many complexation, physicochemical, photochemical, and other studies regarding the interaction of salicylic acid with iron. In this context, the stability constant of its iron complex was determined (log β3 = 36) and found to be similar to that of transferrin and deferiprone, as well its ability to mobilize iron under physiological conditions (log K eff = 19). However, its ability to solubilize polynuclear iron deposits (log K sol = 1), such as those found in ferritin and haemosiderin has been found to be lower than that of deferiprone (log K sol = 9) [100,128].

The interaction of salicylic acid with iron is also utilized for detecting the use of aspirin under different conditions. In particular, the formation of the coloured ferric salicylate complex has been widely used as a diagnostic tool for the detection of aspirin toxicity or in cases of aspirin poisoning [132,133]. However, it should be noted that this method has many limitations and may not be specific to toxicity associated with aspirin. Furthermore, under experimental conditions using spectrofluorimetry, it has been shown that the formation of the ferric salicylate complex can cause quenching of the fluorescence intensity of salicylic acid [131,134].

Another field of investigation in relation to the interaction of salicylic acid with iron is redox activity, which also involves a diagnostic method for the measurement of increased production of hydroxyl radicals in free radical pathology by determining the increased rate of synthesis of 2,3-DHB from salicylic acid in the presence of iron [119,120]. Furthermore, salicylic acid has been shown to have iron chelating and antioxidant activity for example in attenuating gentamicin ototoxicity [135]. In contrast, under different experimental conditions, the redox activity of the iron–salicylate complex is able to induce lipid peroxidation and has been, for example, implicated in atherosclerosis, with profound effects in intravascular and intrahepatic lipids, and also plasma lipoprotein metabolism [136].

The similarity of the biological and clinical activities of aspirin and its initial main metabolite, salicylic acid, is difficult to separate in most cases, especially considering the rapid metabolism of the former to the latter following oral administration [81,82]. This question has become crucial, especially since many clinical findings have suggested that aspirin administration even at low doses (75 mg/day) can prevent colorectal cancer, other cancers, and metastasis [137]. Similar questions also arise as to whether the iron chelation properties of salicylic acid are implicated in the mode of its anticancer activity in colorectal cancer and also other cancers [95,138,139,140].

It is envisaged that further studies on the iron chelation properties of salicylic acid could provide important information related to the pharmacology of aspirin, its iron metabolic effects, and other similar interactions. Further studies are also needed to investigate the influence of iron and the salicylic acid iron complex on the metabolic transformation of salicylic acid in the rate of production of the other aspirin metabolites. In particular, the formation of iron complexes by salicylic acid may influence the production of glucuronide metabolites, similar to the rate of glucurodination of deferiprone observed in iron-loaded patients [97,98].

### 3.3. Interactions of 2,5-Dihydroxybenzoic Acid (Gentisic Acid) with Iron

2,5-Dihydroxybenzoic acid or gentisic acid has been reported to be a natural plant, a mammalian product, and also a metabolite of aspirin [81,82,102]. It is found in many fruits and vegetables and has been reported to have anticancer, anti-inflammatory, antioxidant, antimicrobial, hepatoprotective, neuroprotective, and other beneficial health effects [141]. It has iron-binding properties and has been suggested to act as a mammalian siderophore (chelator) in innate immunity against infection by competing with microbial siderophores [142,143,144].

The iron binding site of 2,5-dihydroxybenzoic acid is similar to that of salicylic acid and consists of the carboxylate ligand and its adjacent hydroxyl ligand (Figure 2). The formation of a 3 chelator:1 iron molar ratio iron complex at physiological pH, with an octahedral structure, is anticipated, similar to the other bidentate iron-chelating drugs and lower-molar-ratio chelator:iron complexes (2:1 and 1:1) under different conditions [100,145]. Furthermore, 2,5-dihydroxybenzoic acid is expected to be more hydrophilic and to form more hydrophilic iron complexes than salicylic acid due to the presence of the extra 5-hydroxyl group.

Several studies have been reported related to the interactions of 2,5-dihydroxybenoic acid with other metal ions in addition to iron [146,147]. However, there are not as many reports on the iron-binding and metabolic effects of 2,5-dihydroxybenzoic acid in comparison to those of 2,3-DHB and salicylic acid. In any case, the overall production of 2,5-dihydroxybenzoic acid is higher than 2,3-DHB from the metabolism of aspirin, and in addition to the increased rate of synthesis from salicylic acid during oxidative stress in the presence of iron [148].

In a marine biology study involving, among other topics, the estimation of the stability constant of the iron complex of 2,5-dihydroxybenzoic acid at physiological pH in seawater, it was shown to be slightly lower (log K eff = 18) than salicylic acid (log K eff = 19) [149]. In another study, the estimation of the level of 2,5-dihydroxybenoic acid in blood was developed using its ability to react with iron, forming an iron complex, which could be identified in blood from its specific physicochemical characteristics [150].

There are hundreds of in vitro and in vivo reports regarding the antioxidant properties of 2,5-dihydroxybenzoic acid. In general, the antioxidant effects of 2,5-dihydroxybenzoic acid appear to have more similarities to salicylic acid than 2,3-DHB. In many studies, comparisons of antioxidant effects with other plant phenolic compounds have been carried out [151]. Some of the antioxidant health benefits of 2,5-dihydroxybenzoic acid include mitigation of drug and radiopharmaceutical toxicity, inhibition of glucose autoxidation involved in the modification of low-density lipoproteins (LDLs) implicated in atherosclerosis in diabetes, iron and copper oxidant toxicity, etc. [152,153,154,155,156,157,158].

The interest regarding the anticancer effects of 2,5-dihydroxybenzoic acid, both as an aspirin metabolite and as a mammalian and plant product, has grown especially since epidemiological studies suggested that aspirin protects against colorectal and other cancers, as well as metastasis. In particular, many research investigators have suggested that 2,5-dihydroxybenzoic acid and other aspirin metabolites are responsible for the anticancer effects. In this context, many studies using various models have suggested that 2,5-dihydroxybenzoic acid can exert anticancer effects via different mechanisms [159,160,161].

### 3.4. Salicyluric Acid as the Major Metabolite of Aspirin in Urine and Interactions with Iron

Salicyluric acid (salicylglycine, or 2-hydroxyhippuric acid) is the major metabolite of aspirin in humans, amounting to about 70% of the metabolites excreted in the urine at doses of 75–100 mg/day [81,82,87,88,89,90,91]. The overall iron-binding capacity of the ACM and related iron metabolic effects are mostly dependent on the concentration of salicyluric acid in biological fluids. In this context, there are many variations in the metabolic transformation of aspirin and the production of salicyluric acid, which depend on many factors such as age, gender, diet, underlying diseases, etc. For example, in one study involving 5 rheumatoid arthritis patients treated chronically with 3.9 g aspirin, which was equivalent to 2.9 g salicylic acid, the mean 24 h urine recovery rates for salicylic acid, 2,5-dihydroxybenzoic acid, and salicyluric acid were 2.9–5.5%, 2.3–2.8%, and 48.9–61.9% respectively [162].

Salicyluric acid is also a natural plant product similar to most of the other ACMs. For example, salicyluric acid has been identified in the urine and serum of normal individuals not taking aspirin. In one study involving 10 individuals not taking aspirin, the levels of salicylic acid and salicyluric acid were estimated in serum using high-performance liquid chromatography (HPLC), with a median concentration of salicylic acid of 0.56 μmol/L and of salicyluric acid of 3.20 μmol/L [163]. Similar results were obtained in another clinical study on non-aspirin users, where the concentration of salicyluric acid in urine was found to be about six times higher than that of salicylic acid [164].

In a comparative clinical study estimating the concentrations of salicylic acid and salicyluric acid in the urine of vegetarians and non-vegetarians, and also patients taking 75 or 150 mg aspirin, it was found that fruit and vegetables are important sources of dietary salicylates. Much higher levels of salicyluric acid (median, 11.01 μmol/24 h) were excreted in the urine of vegetarians than non-vegetarians (median, 3.91 μmol/24), and similarly, overall lower levels for salicylic acid (median, 1.19 μmol/24 h) were found in vegetarians than non-vegetarians (median, 0.31 μmol/24). Interestingly the level of salicylic acid excreted in patients taking 75 or 150 mg aspirin was not different from that found in vegetarians. However, the levels of salicyluric acid excreted in patients taking aspirin was much higher for the 75 mg dose (median, 170.69 μmol/24 h) and the 150 mg dose (median, 165.17 μmol/24 h) than in non-aspirin users [101].

Several studies have been carried out to investigate the affinity of salicyluric acid for iron and other metal ions. The potential ligands involved in salicyluric acid for metal binding are the phenol hydroxyl group and the carbonyl group, as shown in Figure 2. A potential ligand for weaker metal binding is also the distant carboxylate group in salicyluric acid, similar to the carboxylate group of salicylic acid and 2,5-dihydroxybenzoic acid. There have been several studies of iron complex formation with salicyluric acid which suggest that it possesses high affinity for iron [165,166,167]. In particular, in one study, the affinity of salicyluric acid, salicylic acid, and 2,5-dihydroxybenzoic acid for different metal ions was investigated using potentiometric titrations. All three aspirin metabolites were shown to have high affinity for iron (III), aluminium (III), and copper (II) ions, but not for cobalt (II), nickel (II), zinc (II), magnesium (II), or calcium (II) ions. Furthermore, it has been suggested that the iron, aluminium, and copper complexes of these three aspirin metabolites are strong and stable at physiological pH [165].

Several other studies of complex formation with transition metal and other metal ions using salicyluric acid have also been reported [168,169,170,171]. Furthermore, salicyluric acid has also been shown to form mixed complexes with the chelator imidazole and metal ions such as copper and cobalt [168,169]. Similarly, it has been reported to form a terbium binary complex with the macrocycle 1,4,7,10-tetraazacyclododecane-1,7-bisacetate (DO2A), which has been used for the estimation of salicyluric acid in the urine [171].

The iron-binding properties of salicyluric acid and its implication as the major metabolite of aspirin and the ACM with the highest overall iron capacity could increase our understanding of the overall effects on aspirin metabolism and on IDA in chronic aspirin users with different diseases. In this context, further studies are needed, including in vitro, in vivo, and clinical iron mobilization effects, associating the contributory role of salicyluric acid with the overall role of all ACM in IDA.

### 3.5. Interactions of Aspirin (Acetylsalicylic Acid) and Its Glucuronide Metabolites with Iron

In contrast to the ACM, there have not been many reports regarding the interaction of aspirin or, especially, its glucuronide metabolites with iron. In theory, much weaker iron-binding properties are expected from the structural characteristics of aspirin, and more so of its glucuronide metabolites. In this context, and in comparison, for example, with salicylic acid and 2,3-DHB, the phenolic or the carboxylate ligands needed for chelation are blocked by acetate in the case of aspirin, a glucuronide conjugate in the carboxyate ligand in the case of salicylacyl glucuronide, or a phenolic hydroxyl ligand in the case of salicylphenol glucuronide (Figure 1).

However, despite the apparently weak iron-binding properties of aspirin, several investigators have reported the formation and characterization of the aspirin iron complex, other interactions with iron, and also iron metabolism in general. For example, experimental evidence from UV-Vis spectroscopy and HPLC-MS suggested the formation of a coloured aspirin iron complex following interaction with ferrous gluconate [172]. The formation, isolation, and characterization of an aspirin iron complex have also been reported during studies of oxidative damage to the liver mitochondria by aspirin, where the iron complex of aspirin was implicated in the toxicity [173]. In contrast, a mechanism of antioxidant action through the chelation of intracellular low-molecular-weight iron was proposed in cell studies, where aspirin has been shown to protect endothelial cells from oxidative stress damage caused by hydrogen peroxide [174]. Similarly, chelation of iron by aspirin has been suggested to be related to its antioxidant activity in the improvement of cataracts in diabetic models and other models of oxidative damage [175,176].

The interaction of aspirin with many other iron metabolic pathways, including the effects of aspirin on the expression of iron transport and storage proteins and metabolic pathways such as ferroptosis, which is a newly identified programmed cell death involving iron, free radicals and peroxidation of lipids of the cell membrane are attracting the interest of many investigators in almost all diseases [177,178,179,180,181,182,183]. In particular, several studies have suggested that aspirin is involved in the modulation of ferroptosis in many diseases, including chronic kidney disease and cancer [183,184,185,186]. Most importantly, the modulation of ferroptosis by aspirin has been suggested as a mechanism for the treatment of different untreated cancers, anticancer drug resistance, and metastasis [187,188,189,190,191,192,193,194]. Similarly, iron and other transition metal complexes of aspirin and aspirin-based compounds have been suggested as new anticancer agents [195].

The interaction of aspirin with iron and different pathways of iron metabolism, including both iron absorption and iron excretion, needs further investigation. In particular, the interactions of oral aspirin with many other oral iron-containing drug formulations, in addition to ferrous gluconate, may be related to a general mechanism affecting iron absorption, as has previously been shown with other drugs [39,172,196,197,198,199]. Similar effects on iron metabolism may be envisaged from interactions of aspirin with other oral drugs, and also with ascorbic acid, which is used as a supplement or a dietary constituent [200,201,202,203]. Within this context, increased absorption and body intake of copper has been shown in a rabbit animal model using aspirin copper complexes, suggesting that other metal complexes in addition to iron play a role in the absorption of aspirin and the affected metals [39,204]. Similar effects on aspirin absorption, especially on slow-release preparations of aspirin, are also expected from the state of gut microbiota [205]. In contrast to the effects on iron absorption, the iron excretion-related effects of aspirin are very limited considering its rapid metabolism and clearance from the body following oral administration.

No sufficient information is available on the iron-binding properties or related effects of the glucuronide conjugate metabolites of aspirin. In this context, non-significant effects on iron excretion are expected from the glucuronide conjugate metabolites of aspirin, similar to other glucuronide conjugate metabolites of the chelating drugs deferiprone and deferasirox [51].

## 4. Factors Causing Iron Deficiency in Otherwise Normal Individuals Treated Long-Term with Aspirin

Millions of elderly people worldwide, including, for example, 50% of older individuals in the USA, are taking low-dose aspirin (75–100 mg/day) for prophylaxis against many diseases, including cardiovascular and cerebrovascular disease and cancer [83,84,85,206,207,208,209]. In several randomized clinical trials involving long-term users of daily aspirin, iron deficiency was observed in a large portion of participants, which was characterized by decreases in haemoglobin and serum ferritin and was unrelated to gastric bleeding [83,84,85,206,208,210,211,212]. Iron deficiency was not observed either in non-aspirin users or users of other anti-inflammatory drugs [83,84,85].

A major development in relation to low-dose daily aspirin users, which has been suggested following several clinical investigations, was the identification of a decrease in cancer risk and increased survival in cancer patients, which was, according to some investigators, linked to iron reduction caused by aspirin [207,209]. Based on this and other clinical evidence, the USA health authorities recommended the use of low-dose aspirin for the chemoprevention of colorectal cancer in patients 50–59 years of age [191]. The exact mechanism of the anticancer activity and iron reduction in low-dose daily aspirin users is still unclear, and further investigations of aspirin users and cancer are in progress [208,209,210,211,212,213].

In an attempt to solve the puzzle of the cause of iron deficiency in long-term, low-dose daily aspirin users, a major role in the iron-chelating activity of the ACM has been preliminarily proposed considering different parameters, including drug biotransformation and posology effects on iron absorption and iron excretion and overall iron chelation activity [86]. The observation that only a portion of the low-dose, long-term aspirin users develops iron deficiency is another parameter of the puzzle which needs further investigation. This parameter could be considered from previous findings regarding the effect of variety of factors influencing iron balance, iron absorption, and iron excretion [14].

The maintenance of iron balance under normal physiological conditions depends primarily on the effective regulatory control of iron, and especially the overall levels between iron absorption and iron excretion. This balance can be affected by dietary, genotypic, age, iatrogenic, lifestyle, and many other factors [1,2,3,4,9,10,11,12,13,14,15,16,17,18,19,20,21,22,23,24,26,27,28,29,30]. Within this context, there is wide variability in the modes of action of drugs and dietary molecules in relation to iron absorption and iron excretion. For example, oral deferoxamine prevents iron absorption, and subcutaneous deferoxamine increases iron excretion mostly in the urine, but also in the faeces. On the other hand, oral deferasirox increases iron absorption and iron excretion in the faeces. Similarly, maltol increases iron absorption, but does not significantly affect iron excretion. The mode of action of oral deferiprone is also different, since it does not significantly affect iron absorption, but increases urinary iron excretion [46,47,50,51]. Another important parameter affecting both iron absorption and iron excretion in relation to drugs with chelating potential, including aspirin and metabolites, are the individual rate of ADMET parameters in each patient, as well as other parameters, such as the iron load, availability, and concentration of iron available for chelation, as well as the length of treatment [53,54,55,56,57,58,59,60,214].

The effects of orally administered aspirin in relation to iron absorption and vice versa have not yet been fully investigated. However, considering the pharmacological and metabolic profile of orally administered aspirin and its partial conversion to salicylic acid in the GIT, it is likely that only a small reduction in dietary iron absorption is caused by both aspirin and salicylic acid binding of dietary iron, which is especially feasible if aspirin is taken during meals or pharmaceutical formulations containing iron are consumed [172].

A different mechanism of iron excretion involving aspirin and ACM is anticipated. In this case, most of the orally administered aspirin seems to be absorbed and metabolically converted to salicylic acid and the other metabolites, with, overall, more than 70% of aspirin associated with the biotransformation to ACM capable of mobilizing iron from different iron pools and causing an increase in iron excretion [7,51,86]. In particular, the mode of chelating activity of aspirin and the ACM resembles the widely used iron chelation combination therapy, where the combination of chelating drugs is more effective in increasing iron excretion than any monotherapy [55,56,57,86,108].

There are several other parameters to be considered regarding the concentration and efficacy of the ACMs. In contrast to chelating drugs such as oral deferiprone and deferasirox, which are rapidly transformed to glucuronide metabolites with no chelating properties, the ACMs are excreted in the urine with no further metabolic transformation (Figure 2) [80,81,82,86]. The drug posology administered, as well as the drug and, in this case, ACM concentration in the blood, are important parameters in determining the level of iron excretion during chelation therapy [86]. For example, several metabolic studies involving iron balance and deferiprone have shown that, in the vast majority of cases of iron-loaded patients, deferiprone is mostly metabolised and excreted as its glucuronide conjugate metabolite, with only 3–20% excreted in the urine as deferiprone iron complex [97,98]. In general, the level of iron excreted by deferiprone and other chelating drugs depend on the drug dose and the iron load of the patient [56,57,58,59,215,216]. Much lower doses of deferiprone and other chelating drugs are used in non-heavily iron-loaded thalassaemia patients, as well as different non-iron-loaded categories of patients with neurodegenerative and other diseases [53,54]. In such cases, regular monitoring of iron parameters is essential to prevent iron deficiency and other toxicities [52,56].

Limited information is available on iron balance studies and the general comparison between iron absorption and iron excretion in otherwise normal elderly individuals taking aspirin and non-aspirin users in the absence of major gastric bleeding. However, in several randomised clinical trials, the use of aspirin has been considered as the major cause or one of the causes of iron deficiency [83,84,85,206,207,208]. A general estimation of the level of changes in iron balance caused by ACM could be considered based on available data from dietary iron absorption levels and iron excretion caused during chelation therapy.

In general, the maintenance of the body’s iron balance is accomplished through the daily absorption of about 2 mg of iron from a typical Western diet and the equivalent daily amount of body iron excretion or loss [1,2,3,13,14,15,16,47]. Despite the fact that iron balance studies have not been carried in individuals using aspirin or ACM, some information is available from related studies on the bidentate chelators deferiprone and 2,3-DHB. In this context, low doses of deferiprone at 0.5 g and 1.0 g of about 7.5 mg/kg and 15 mg/kg caused net iron increases of approximately 0.2 mg and 0.7 mg, respectively, in the urine of a myelodysplasia patient with serum ferritin of 920 μg/L [217]. Low increases in iron excretion (2.7–6.9 mg) were also observed in four myelodysplasia patients (40–75 kg, serum ferritin 750–2040 μg/L) who received 2 g of deferiprone (27–50 mg/kg), and in thalassaemia patients (6.5–22.8 mg iron) who received 1–1.5 g of deferiprone (23–33 mg/kg) [218]. Much lower levels of iron excretion have been reported in normal volunteers and patients with normal iron stores when treated with deferiprone [56,57,58,59,97,98]. For example, administration of deferiprone at 35 mg/kg in a normal volunteer (serum ferritin 210 μg/L) caused a net increase of 0.84 mg of iron [97]. Only 154 mg of a 3.0 g dose of deferiprone was associated with the 0.84 mg increase in urinary iron excretion, while the remaining was excreted as a deferiprone glucuronide [97]. As previously mentioned, the increased iron excretion caused in thalassaemia patients using 2,3-DHB at 25 mg/kg/day was, on average, 4.5 mg iron, and this is expected to be much lower in normal individuals [106]. However, in the case of low-dose aspirin, the overall iron chelating capacity of the ACM, as estimated from the urinary levels, could be about 50 mg, which might be sufficient for a smaller increase in urinary iron excretion [97,106]. It is therefore envisaged that, despite the low level of daily net iron excretion increases by 2,3-DHB and of the other ACM in low-dose aspirin users, the overall incremental iron excretion increases induced over months and years by ACM could be sufficient to cause iron deficiency.

The observation that only a minority (e.g., of about 23% [83]) of the low-dose, long-term aspirin users develop IDA is another parameter of the puzzle of the interaction with iron which needs further investigation. However, considering that the same or similar dilemmas appear in relation to the incidence of IDA in only some sections of the vegetarian population, other parameters should be considered and included in the equation of iron imbalance. In particular, it is suggested that the portion of the low-dose, long-term aspirin users developing IDA may be associated with additional causes or activities, such as those who are also vegetarians or who have dietary habits with vegetarian meals low in iron, or those who are involved in blood donation or intensive sport [26,27,28].

Other factors implicated in iron deficiency affecting aspirin users are dietary habits using food containing phosphates, tannic acid, and other components which inhibit iron absorption [34,35,36,37,38]. In particular, food containing salicylates, including the ACM salicylic acid, 2,5-dihydroxybenzoic acid (b), 2,3-DHB, and salicyluric acid, may inhibit iron absorption and increase iron excretion [163,164]. However, the overall effect of dietary salicylates on iron deficiency is much lower than in aspirin users because of the low content in vegetarian meals in comparison to the ACMs formed from the metabolism of low doses of aspirin [101].

It appears that many factors and mechanisms may participate in the development of iron deficiency in the section of individuals using long-term, low-dose daily aspirin. However, it also appears that, in the majority of cases of aspirin users, any iron losses due to increased iron excretion by ACM and other causes are compensated for by dietary iron sources, thus maintaining iron balance in a mechanism similar to the gradual restoration of iron balance in blood donors (Table 1).

It is therefore suggested by direct and indirect evidence that a primary role in the cause of iron deficiency in aspirin users is played by the ACM. In such cases, the chronic administration of daily doses of aspirin is likely to increase the overall iron excretion in small increments each time, which is caused by the combinatory iron mobilization effect of the ACM formed by aspirin. This effect and the consumption of a vegetarian diet low in iron are likely to be the main contributors to iron deficiency (Table 1). Smaller contributions to iron deficiency may also involve the possible inhibition of iron absorption in GIT by aspirin and salicylic acid, as well as dietary salicylates. Similarly, the dietary salicylates salicylic acid, 2,5-dihydroxybenzoic acid, 2,3-DHB, and salicyluric acid may also cause slight increases in iron excretion and contribute to the overall loss of iron in long-term, low-dose aspirin users.

## 5. Future Studies and Limitations on the Role of Aspirin in Iron Deficiency and Other Clinical Conditions

There are many different limitations to the mode of action and therapeutic activity of aspirin, including the implications of iron chelation and other effects of the ACMs (Table 1). Some of the limitations and effects are related to differences in the doses and formulations of aspirin use, as well as the underlying conditions, dietary habits, ADMET, and other characteristics of aspirin users. For example, the widely used low doses of 75 mg and 100 mg aspirin are arbitrary in most cases, since they are intended for people of 70 kg, and those of higher weights or obese individuals may not achieve the desired therapeutic aspirin threshold [219,220]. Furthermore, in some cases, the therapeutic benefits of low-dose aspirin for cancer and cardiovascular diseases are usually observed after 5 years or more of daily use [221]. Similar effects and implications are expected from the posology and time period of aspirin use on IDA, since the iron-mobilizing and other effects of ACM are dependent on the concentration and rate of mobilization (Table 1) [219].

Further investigations into the role of aspirin and each of the ACMs are needed in relation to iron deficiency and other diseases of iron metabolism, similar to those used for the development of chelators for the treatment of iron overload and chelator iron complexes for the treatment of iron deficiency [14,51,100,108,222]. The chemical interactions with iron, including the further characterization and stability of the iron complexes, as well as in vitro, in vivo, pharmacological, toxicological, and clinical studies, could provide sufficient information on the overall impact of ACM on iron excretion and the cause of iron deficiency in aspirin users [51,100,108,222]. Many such studies, including clinical trials on iron balance related to increases in iron excretion, which have already been carried out with 2,3-DHB, are also needed for the rest of the ACMs [105,106,107].

The study of the effects of aspirin and ACMs on other essential metal ions in addition to iron, including calcium, zinc, and copper, are also important for possible implications on the general mode of action and toxicity of aspirin [218,223,224]. For example, increases in the excretion of these metal ions or redistribution in the body can result in toxic side effects similar to those observed with DTPA and also other chelating drugs [60,225]. Different metabolic and toxic side effects are also expected from the interactions of aspirin and ACM with proteins and metabolic pathways related to these essential metals.

The relationship of aspirin’s anti-inflammatory activity and its antioxidant potential has not yet been clarified, despite some studies which have previously suggested there may be a link between these two processes [173,174,175,176]. Furthermore, the redox effects of all of the ACMs have not been thoroughly investigated in relation to their iron-binding potential and the Fenton reaction, where iron is the catalytic centre of the formation of very reactive and toxic hydroxyl radicals [116,118,119,148,149,152,154,155,156,157]. In addition, previous studies have shown that deferoxamine, deferiprone, and similar chelators inhibit free radical reactions and the activity of the iron-containing proteins cyclo-oxygenase and lipoxygenase, as well as their metabolic by-products [118,226,227,228]. The mode of action of chelation in this case was suggested to be the reduction in available iron for the turnover of cyclo-oxygenase and lipoxygenase activity [226,227,228]. In this context, further studies are needed to investigate the partial inhibition of cyclo-oxygenase and lipoxygenase through iron chelation in relation to the overall anti-inflammatory activity of aspirin and ACM, as well as the implications of this inhibitory activity in other diseases.

Oxidative stress toxicity damage, which is mostly catalysed by iron catalytic centres, has been identified in almost all diseases in the context of free radical pathology, including those involving organ and tissue damage, cancer, neurodegenerative diseases, and many other conditions [43,118,226]. Chelators, which can have access and bind iron at the iron’s catalytic centre, can effectively inhibit this toxicity [43,226]. In this context, the interactions of the ACMs with the iron catalytic centres involved in this form of toxicity could provide further evidence on the therapeutic activity of aspirin and ACM and other health implications in aspirin users.

A major unexplored area of great importance in pharmacology and medicine is the effects of aspirin and ACM, as well as their iron complexes, on ferroptosis, which is a recently identified type of programmed cell death based on iron-catalysed lipid peroxidation damage of cell membranes [179,180,181,182,183,184]. Ferroptosis has been identified in almost all diseases, including cancer and neurodegeneration [179,180,181,182,183,184,185,186,187,188,189,190]. Several preliminary investigations have already implicated aspirin in the process of ferroptosis via different mechanisms [192,193,194]. In this context, the interaction of aspirin, ACMs, and their iron complexes in ferroptotic cell death may have wider implications in the therapeutic outcome of aspirin in cancer and many other related diseases.

The wide posology selection of aspirin for use in different diseases, which can range from about 50 mg to 3.6 g per day, provides much-needed information regarding the pharmacology, toxicity, and iron chelation efficacy of the ACMs [64,65,66,67,68,229,230,231,232,233]. The prospect of clinical testing of aspirin and ACM at the maximum range of posology for increasing iron excretion in thalassaemia and other iron-loaded diseases, similarly to the clinical studies previously carried out with 2,3-DHB, could further increase the range of clinical applications of aspirin and ACM in different diseases. In particular, such information could increase the prospects of clinical application of aspirin and ACM in colorectal and other cancers, and in the detoxification of some other xenobiotic metals, where higher concentrations of chelating drugs such as deferiprone and EDTA compared to those achieved by low-dose aspirin ACM will be required for effective treatments [223,225,226,234].

Overall, further in vitro, in vivo, and clinical studies are required for selecting aspirin and/or one or more of the ACMs for clinical use in different conditions. Similarly, studies on the selection of the appropriate posology of aspirin and ACM will be necessary for achieving maximum efficacy and low toxicity in each of the associated clinical conditions.

## 6. Conclusions

Several randomized, double-blind clinical trials of long-term, low-dose aspirin users involving otherwise healthy elderly patients, have shown that a large percentage of such individuals develop IDA in the absence of major gastric bleeding. The cause of iron deficiency has not been identified. In this review, a significant amount of evidence is presented suggesting that most of the metabolites of aspirin have iron-chelating properties, and one of them, 2,3-DHB, has been shown to cause increases in iron excretion in iron-loaded patients. In particular, it has been suggested that the ACMs salicyluric acid, salicylic acid, 2,5-dihydroxybenzoic acid and 2,3-DHB can all act jointly as a form of combination iron chelation therapy and enhance the overall iron excretion in small increments over time, which can result progressively, over many months or years, in iron deficiency in chronic aspirin users. Many other factors may also contribute to iron deficiency in this category of long-term, low-dose elderly aspirin users and especially in cases of individuals who are consuming vegetarian diets, who are low in iron, or who are, for example, involved in intense sport activities. In contrast, it is likely that, in the majority of long-term, low-dose aspirin users who are non-vegetarians, sufficient iron could be absorbed from normal non-vegetarian diets to compensate for the increase in iron excretion caused by the ACM.

Further in vitro, in vivo, and clinical investigations are required for the characterization of the iron-binding effects of the ACM, including iron balance studies and interactions with proteins and metabolic pathways involving iron. Similar investigations are also needed in relation to the therapeutic effects of aspirin and ACM observed in different conditions, including cancer, neurodegeneration, and iron overload, as well as in diseases involving ferroptosis and other diseases related to free radical pathology.

## Figures and Tables

**Table 1:** The role of aspirin chelating metabolites on iron deficiency and the influence of other factors.

**A. The properties and iron binding effects of the aspirin-chelating metabolites**
**Molecular properties of the aspirin-chelating metabolites** All aspirin metabolites are of low molecular weight, with the smallest being salicylic acid. The ACMs are negatively charged at physiological pH due to the presence of a carboxylate group. All the aspirin metabolites are cleared through the kidneys and eliminated through the urinary route.
**Achievement of high concentration of the aspirin-chelating metabolites with high iron capacity** Almost 70% of aspirin is metabolized to ACM, with salicyluric acid predominating (>65%) over the other metabolites. The ACMs have sufficiently long half-lives and can reach optimal concentrations for the mobilization of iron. The combined concentration of the ACM has a high iron binding capacity equivalent to that of iron-chelating drugs.
**Evidence of the high affinity for iron by the aspirin-chelating metabolites** The iron complex formation of the ACM, with specific physicochemical characteristics in each case, indicates a high affinity for iron, with high stability constants and the ability to mobilize iron. For example, the iron stability constant of salicylic acid (log β3 = 36) is similar to that of the iron-chelating drug deferiprone.
**Iron mobilizing efficacy of the aspirin-chelating metabolites in preclinical studies** The ACMs have been shown to mobilize iron from aqueous solutions, ferritin, cells, and different animal species.
**Iron mobilizing efficacy of the aspirin-chelating metabolites in clinical studies** The ACM 2,3-dihydroxybenoic acid has been shown in several clinical trials to be effective in increasing substantially iron excretion in iron-loaded thalassaemia patients. Smaller amounts of iron excretion are expected in aspirin users with normal iron stores.
**Cumulative effects on iron mobilization by the aspirin-chelating metabolites** The combinatory effect of the ACM in increasing iron mobilization and iron excretion resembles the combination chelation therapy, which is more effective than monotherapy. Despite the small incremental increases in daily iron excretion caused by ACM, total iron loss over months and years could lead to iron deficiency.
**B. Other factors influencing iron deficiency in low-dose daily aspirin users**
**The influence of dietary iron on iron balance in elderly individuals using low-dose aspirin daily** In the majority of cases of low-dose daily aspirin users, in the absence of major gastric bleeding, iron balance is restored gradually from dietary iron, similarly to blood donors. Iron deficiency is observed only in a minority of cases of low-dose daily aspirin users in the absence of major gastric bleeding. It is likely that such individuals are vegetarians or receive mostly vegetarian meals with insufficient absorbable content.
**Additional factors to dietary iron contributing to iron deficiency in daily low-dose aspirin users** There are additional factors involved in insufficient dietary iron body intake which can contribute to iron deficiency in daily low-dose aspirin users. These include individuals who are blood donors or engaged in heavy sport activities, as well as malabsorption of iron due to complications in the GIT. Minor undetected blood loss may also contribute to iron deficiency.
**Cumulative effects contributing to iron deficiency in daily low dose aspirin users** One or more of the aforementioned factors, i.e., low dietary iron intake, blood donation, heavy sports, or minor bleeding, may contribute to the overall increases in body iron loss. The contributory effect of each of these factors is different for each individual.

ACM: aspirin-chelating metabolites. GIT: gastrointestinal tract.

## Abbreviations

ACM	chelating metabolite
ADMET	absorption, distribution, metabolic, elimination, toxicity
Aspirin	acetylsalicylic acid
COX	cyclo-oxygenase
2,3-DHB	2,3-dihydroxybenzoic acid
DO2A	1,4,7,10-tetraazacyclododecane-1,7-bisacetate
DTPA	diethylenetriaminepentaacetic acid
EDTA	ethylenediaminetriacetic acid
GIT	gastrointestinal tract
HPLC	high-performance liquid chromatography
IDA	iron deficiency anaemia
